# Unlocking the potential of electronic blood transfusion systems: Implementation insights from NHS hospitals in England

**DOI:** 10.1111/bjh.20198

**Published:** 2025-06-10

**Authors:** F. Tomini, S. Staples, H. Evans, H. Farabi, M. F. Murphy, L. Green, P. Dhiman, G. Fabiano, A. J. R. Palmer, L. von Neree, L. Sherliker, S. J. Stanworth

**Affiliations:** ^1^ Wolfson Institute of Population Health Queen Mary University of London London UK; ^2^ Blood Stocks Management Scheme NHS Blood and Transplant Filton Bristol UK; ^3^ NIHR Blood and Transplant Research Unit in Data‐Driven Transfusion Practice, Nuffield Division of Clinical Laboratory Sciences, Radcliffe Department of Medicine University of Oxford Oxford UK; ^4^ NHS Blood and Transplant Oxford University Hospitals NHS Foundation Trust and University of Oxford Oxford UK; ^5^ Blizard Institute Queen Mary University of London London UK; ^6^ NHS Blood and Transplant Barts Health NHS Trust London UK; ^7^ Nuffield Department of Orthopaedics, Rheumatology, and Musculoskeletal Sciences University of Oxford Oxford UK

**Keywords:** digital technology, electronic blood transfusion systems, health IT integration, implementation factors, NHS England

## Abstract

Electronic blood transfusion (EBT) systems have the potential to significantly enhance patient safety and healthcare efficiency. Although national guidelines recommend their implementation, widespread adoption requires addressing gaps in evidence regarding cost‐effectiveness and clinical impact. This study assessed EBT implementation in English hospitals via a 2023 survey focusing on three key components: (i) electronic blood fridges (EBFs) for traceability and stock management, (ii) electronic blood ordering systems with clinical decision support (CDSS) and (iii) bedside patient identification (PID) systems. The survey also examined EBT integration with electronic health records (EHRs) and inter‐hospital record linkages. Among 206 surveyed hospitals, 114 responded, resulting in a 55.6% response rate. Of the responding sites, 76 (67.3%) had implemented at least one EBT component. EBFs were the most adopted technology (65 sites; 57.5%), followed by bedside identification systems (37 sites; 32.7%). Advanced systems like CDSS were implemented in only 16 sites (14.2%). Barriers to adoption included financial constraints, limited senior management engagement and technical challenges. Our results show variability in EBT system adoption across NHS England hospitals. There is a pressing need for cost‐effectiveness analyses to support investment decisions, while evidence of clinical effectiveness is needed to justify advanced EBT systems and overcome organisational barriers.

## Graphical abstract

To assess the current implementation of electronic blood transfusion (EBT) systems, associated barriers, and future intentions, we conducted a national survey among 206 NHS hospitals in England, with a 55.6% response rate (114 hospitals). The most frequently implemented component was electronic blood fridges (57.5%), followed by bedside patient identification (32.7%) and electronic blood ordering systems with clinical decision support (14.2%). Key barriers to broader implementation included financial constraints, limited senior management support and technological challenges. Further evaluation and cost‐effectiveness studies are needed to support evidence‐based investment and adoption of these systems.

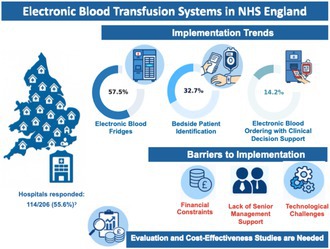

## INTRODUCTION

Advances in healthcare technology offer transformative opportunities to enhance operational efficiency and improve patient outcomes, and this certainly applies to transfusion practice in hospitals. Electronic blood transfusion (EBT) systems integrate various technologies designed to streamline and ensure safe processes such as patient identification, blood sample collection for compatibility testing, blood ordering and issue, as well as administration and tracking of blood units transfused. These technologies have demonstrated substantial potential to optimise blood usage, reduce errors and support cost‐effective management of transfusion services.^1^, ^2^, ^3^, ^4^, ^5^, ^6^, ^7^, ^8^, ^9^ Core features of EBT systems include barcode‐based identification for patient and blood product matching, remote issue of blood units, blood stock management and clinical decision support system (CDSS) that aid in decision‐making for the appropriate use of blood.^10^, ^11^ These technologies not only modernise transfusion practices but also address challenges such as minimising wastage, improving traceability and enhancing compliance for patient safety.^5^, ^12^, ^13^, ^14^

The latest report and recommendation from the infected blood inquiry^15^ underscored the importance of implementing EBT systems to improve transfusion safety and ensure accountability in transfusion practices. It highlighted the need for targeted funding to support the adoption of EBT systems across hospitals, particularly for foundational components like electronic patient identification (PID) and traceability systems. The inquiry also recommended the development of robust health economic evidence to demonstrate the cost‐effectiveness of EBT technologies, which would facilitate their prioritisation in healthcare budgets. These recommendations align with the findings from recent studies that emphasise the role of EBT systems in enhancing patient safety, minimising transfusion‐related errors and optimising resource use.^1^, ^2^, ^3^, ^4^, ^8^

In light of these recommendations, understanding the current implementation landscape is critical to guiding the future development and sustainable nationwide adoption of EBT systems. This study describes the implementation status of EBT systems across hospital services in England, highlighting adoption trends, barriers to implementation and the impact of these systems on transfusion practice. The findings, which assess the current landscape, aim to inform strategies for addressing challenges and promoting broader adoption of EBT systems to enhance transfusion safety and operational efficiency.

## METHODS

Between June and September 2023, a structured electronic data collection form was developed, piloted and distributed to 206 hospital sites in England to evaluate the implementation of EBT systems.

All the surveyed hospitals were registered under the Blood Stocks Management Scheme (BSMS). The BSMS conducts blood inventory practice surveys among 258 hospital sites served by NHS Blood and Transplant (NHSBT).^16^ For this survey, hospitals classified by the BSMS as ‘Very Low’ red blood cell (RBC) users—reporting annual RBC issues between 80 and 350 units—were excluded. This accounts for the difference between the total hospitals registered with BSMS and those surveyed (206). The final list comprises the hospital sites that responded independently or as part of an NHS trust, explaining variation in the number of hospitals and trusts represented.^17^ This study focuses on hospital‐level analysis, referred to here as ‘sites.’

The research team^18^ developed and pretested a data collection tool focusing on three key components of EBT systems: (i) EBFs, which support blood traceability and stock management^3^, ^19^; (ii) electronic blood ordering systems, which may incorporate CDSS to guide appropriate blood requests^20^, ^21^; and (iii) bedside PID systems, used for sampling, transfusion verification and tracking.^4^, ^5^ The tool also collected data on EBT capabilities, such as linking blood records with patients' electronic health records (EHR) and connecting transfusion records between hospitals to access historical information about blood groups, antibodies, transfusion reactions and special transfusion requirements. Respondents were asked to rank barriers to implementation, including financial constraints, limited senior management engagement, technical integration challenges and insufficient training resources. Barriers were ranked by significance and priority areas for improvement. Finally, respondents indicated how they use EBTs for reporting, monitoring, auditing and traceability.^22^

Respondents were requested to provide the names of their EBT suppliers, the timelines of adopting EBTs, the extent of the clinical services covered by each element of their EBT and details of the challenges faced during the implementation process. They were also asked to share their plans for expanding or upgrading EBT systems.

Data were analysed using descriptive statistics and statistical tests (Chi‐squared and Fisher's Exact)^23^, ^24^ to explore group associations. Additional analysis assessed variation in site participation using geographic and organisational data from the BSMS^16^ and the NHS Statistics Database,^25^ matched at the hospital or trust level with our survey respondents.

## RESULTS

### Survey respondents

We received responses from 114 (55.6%) out of the 206 surveyed hospital sites, representing 91 of the 152 surveyed NHS Trusts in England (59.9%). Table 1 compares the response rates of hospitals stratified by their red blood cell (RBC) usage categories as classified by the BSMS. Hospitals with ‘very high’ RBC use had the highest response rate (67.7%), followed by those with ‘moderate’ RBC use (63.6%) and ‘high’ RBC use (54.0%). In contrast, hospitals categorised as ‘low’ RBC users demonstrated the lowest response rate (27.0%).

**TABLE 1:** Respondents versus non‐respondents and EBTs in place.

Category	Respondents (*N*, %)	Non‐respondents (*N*, %)	Statistical tests
Total trusts	91/152 (59.9%)	61/152 (40.1%)	
Total hospital sites	114/206 (55.3%)	92/206 (44.7%)	
BSMS 2023 categories for blood use^a^			
Very high	21/31 (67.7%)	10/31 (32.3%)	*χ* ^2^ = 16.42, *p* = 0.001 Fisher's exact: *p* = 0.001
High	27/50 (54.0%)	23/50 (46.0%)	
Moderate	56/88 (63.6%)	32/88 (36.4%)	
Low	10/37 (27.0%)	27/37 (73.0%)	
EBTs elements in place (latest year, 2023)			
At least one EBT element	76/113 (67.3%)	‐	
No EBT in place	37/113 (32.7%)	‐	
Missing/not reporting	1/114 (0.9%)	‐	
System statuses (latest year, 2023)			
PID: Sample labelling	29/113 (25.7%)	‐	
PID: Blood administration	37/113 (32.7%)	‐	
Blood fridge: No remote issue	65/113 (57.5%)	‐	
Blood fridge: Remote issue	9/113 (8.0%)	‐	
Blood order: No CDSS	3/113 (2.7%)	‐	
Blood order: With CDSS	16/113 (14.2%)	‐	
Linkage with EHRs	32/113 (28.3%)	‐	
Linkage of records within/between hospitals	33/113 (29.2%)	‐	
Traceability	47/113 (41.6%)	‐	

Abbreviations: CDSS, clinical decision support system; EBT, electronic blood transfusion; EHR, electronic health record; PID, patient identification.

^a^ Blood Stock Management Scheme (BSMS) collects data on red blood cell (RBC) unit issues, stock levels and wastage in hospitals. The BSMS ‘user categories’ are used to organise hospital responses according to the number of annual RBC issues. Hospitals with over 10K RBC issues per year are considered ‘very high’, >6000 and ≤10 000 ‘high’, >3000 and ≤6000 ‘moderate’, >500 and ≤3000 ‘low’ and hospitals with ≤500 RBC issues or below as ‘very low’.

Statistical analysis revealed significant differences in engagement between RBC usage categories. The chi‐squared test (*χ* ^2^ = 16.42, *p* = 0.001) and Fisher's exact test (*p* = 0.001) highlighted that sites in higher RBC usage categories were significantly more likely to respond to the survey compared to those in lower categories.

We conducted supplementary analyses using publicly available data to assess whether organisational or geographical factors influenced survey participation (see Tables [Link], [Link]). Comparisons by service type, bed capacity, NHSBT Stock Holding Units (SHU) affiliation and distance to SHU showed no significant differences between respondents and non‐respondents, supporting the representativeness of the sample.

### EBTs implementation

Table 1 shows that EBFs without remote issue were the most widely adopted component (57.5%, 65 sites). In contrast, more advanced features, such as blood ordering with CDSS (14.2%), without CDSS (2.7%) and EBFs with remote issue (8.0%), had much lower adoption rates. Moderate uptake was observed for PID systems used during blood sampling (25.7%) and administration (32.7%), as well as for data linkage capabilities, including integration with EHRs (28.3%) and linkage of records within or between hospitals (29.2%).

The implementation of EBT components varied significantly among sites. Only two hospitals reported full implementation of all elements of electronic transfusion systems (Table S5). The adoption of EBFs without electronic remote blood issue began as early as 1998, emphasising early efforts to improve blood traceability, storage and availability at the time of collection. More advanced technologies, like EBT ordering systems incorporating CDSS, were introduced as early as 2008, with adoption rates picking up only around 2019 (Table S6).

**FIGURE 1:**
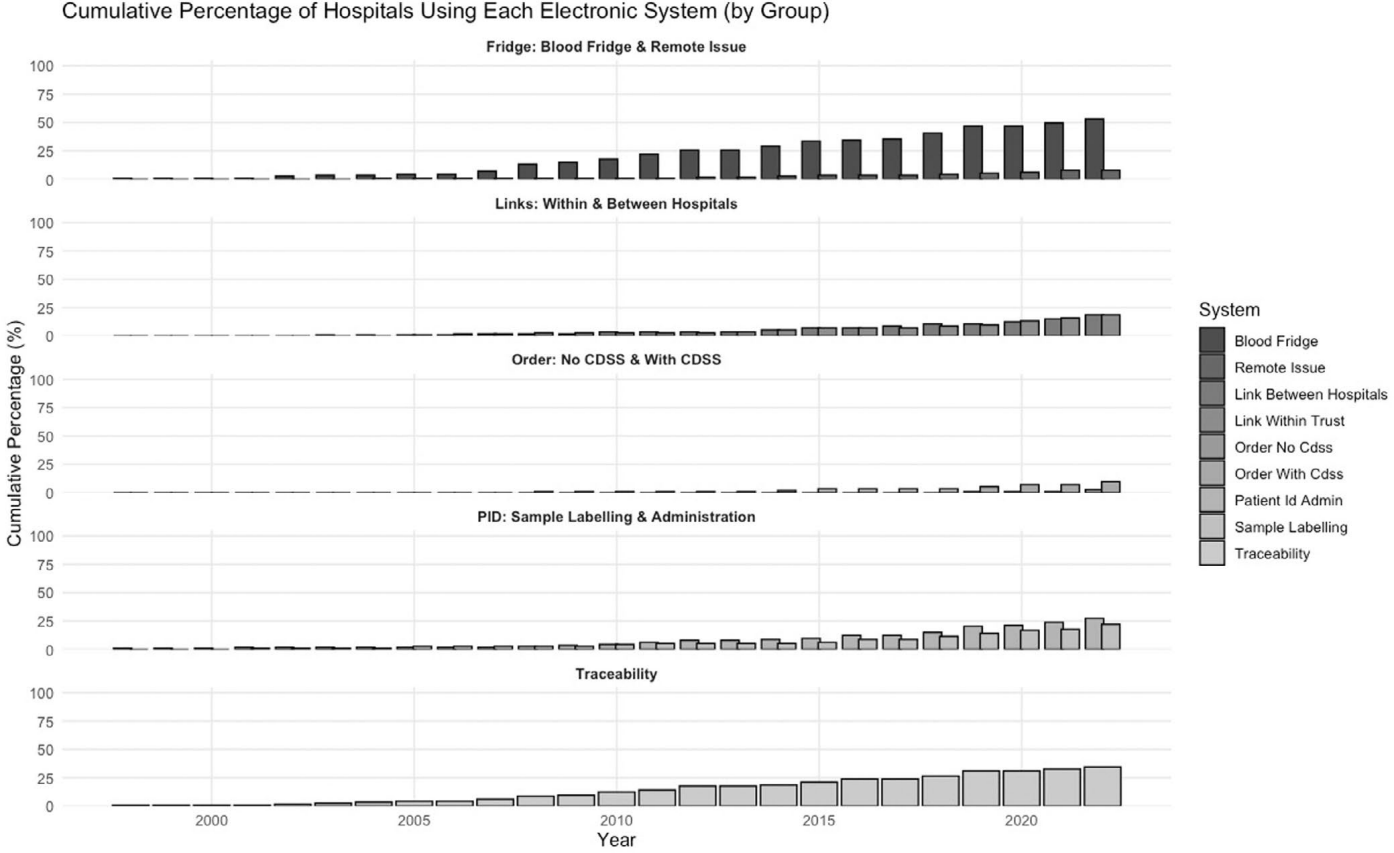
Timeline of implementation of the electronic blood transfusions (EBTs) among the surveyed hospitals. Cumulative percentage of NHS hospitals using each component of EBT systems over time. Bars represent cumulative adoption of EBTs for: Electronic blood fridges (with and without remote issue), the linkage of electronic transfusion records with patient records and the linkage of transfusion data between hospitals of the same trust, blood ordering systems (with and without clinical decision support—CDSS), patient identification systems for blood administration and sample labelling, and blood traceability systems. Data are based on the reported year of implementation and highlight the staggered and variable rollout of EBT components across NHS England hospitals over the years.

Figure 1 illustrates a consistent upward trend (as a % of all responding hospital sites) in adopting various EBT components among surveyed hospitals, especially from 2010 to 2023. While the overall trajectory is positive, the adoption rates for advanced EBT systems, including CDSS and electronic remote blood issue, remain low. This indicates significant potential for growth and broader implementation throughout the NHS.

Table S7 provides further insights into the proportion of total transfusions utilising key EBT components in hospitals, highlighting both the variability in their implementation and the rates of full adoption. EBFs show the highest rate of full implementation, with 48.7% (55 hospitals) using them across all transfusions. Traceability procedures and record linkage (within/between hospitals) follow, at 33.6% (38 sites) and 28.3% (32 sites) respectively. Meanwhile, linkage with EHRs is fully implemented by 25.7% (29 sites).

In contrast, fewer hospitals have fully adopted bedside patient identification (18.6%, 21 sites) or sample taking and labelling (11.5%, 13 sites) (Table S7). Advanced electronic blood ordering systems, including those with CDSS, also remain limited, with 11.5% (13 sites) achieving full implementation. Remote blood issue systems show the lowest rate of full adoption at 2.7% (3 sites).

#### Priorities and barriers

The analysis of self‐declared priorities for future implementation (Table 2) highlights varying levels of emphasis across different EBT components. Bedside electronic PID systems (56 sites, 56.0%) and automated blood collection systems (43 sites, 50.0%) emerged as top priorities, followed by linkage of transfusion records within or between hospitals (44 sites, 47.3%). In contrast, CDSS (28 sites, 30.4%) and EHR linkage (25 sites, 25.0%) were less frequently prioritised.

**TABLE 2:** Priority of implementing EBT components.

Priority	Bedside electronic management system	Computerised decision support	Automated blood collection system	Linkage with EHRs	Linkage of records within/between hospitals
High/very high (rate 1 and 2)	56/100 (56.0%)	28/92 (30.4%)	43/86 (50.0%)	25/100 (25.0%)	44/93 (47.3%)
Moderate (rate 3)	14/100 (14.0%)	23/92 (25.0%)	15/86 (17.4%)	25/100 (25.0%)	18/93 (19.4%)
Low/very low (rate 4 and 5)	30/100 (30.0%)	41/92 (44.6%)	28/86 (32.6%)	50/100 (50.0%)	31/93 (33.3%)

Stat. test on differences between priority groups (very high/high versus moderate versus low/very low)					
*χ* ^2^ (*p*‐value)	6.77 (0.034)	4.42 (0.109)	5.69 (0.058)	8.67 (0.013)	4.89 (0.087)
Fisher's exact	(0.036)	(0.114)	(0.060)	(0.014)	(0.091)

Abbreviations: EBT, electronic blood transfusion; EHR, electronic health record.

Statistical analyses reveal significant differences in prioritisation for bedside EBTs (*p* = 0.034) and EHR linkage (*p* = 0.013). These findings indicate variability in how hospitals perceive the importance and readiness for adopting specific EBT components. However, not all components showed statistically significant differences, suggesting that prioritisation may vary depending on hospital‐specific factors and existing infrastructure.

Lack of funding from senior management was the most significant barrier (Table 3), with 70.7% of respondents rating it as very high or high (*p* < 0.001). Senior management engagement followed, with 42.6% identifying it as a major challenge. Time and resource limitations were also widely cited (42.3% very high/high; *p* = 0.049). While less prominent, barriers such as limited understanding of patient benefits (25.5%) and cost‐savings (22.6%) also showed significant differences across response categories (*p* = 0.017 for cost‐savings).

**TABLE 3:** Barriers to implementing EBT components.

Barriers	Incomplete understanding of patient benefits	Incomplete understanding of cost‐savings	Lack of engagement from senior management	Lack of funding from senior management	Lack of time or resources from transfusion teams
Barriers: Very high/high	24/94 (25.5%)	21/93 (22.6%)	40/94 (42.6%)	70/99 (70.7%)	41/97 (42.3%)
Barriers: Moderate	20/94 (21.3%)	20/93 (21.5%)	27/94 (28.7%)	7/99 (7.1%)	20/97 (20.6%)
Barriers: Low/very low	50/94 (53.2%)	52/93 (55.9%)	27/94 (28.7%)	22/99 (22.2%)	36/97 (37.1%)

Stat. test on differences between priority groups (priority very high/high versus moderate versus low/very low)					
*χ* ^2^ (*p*‐value)	6.77 (0.034)	8.23 (0.016)	5.14 (0.077)	28.53 (0.000)	7.96 (0.047)
Fisher's exact	(0.036)	(0.017)	(0.079)	(0.000)	(0.049)

Abbreviation: EBT, electronic blood transfusion.

#### EBTs, data feedback to physicians, traceability and the level of staff

The analysis of feedback and data availability to clinicians (Table 4) showed that routine availability of blood usage data is significantly higher in sites using EBTs (11 sites, 9.8%) compared to non‐EBT hospitals (0 sites, 0.0%) (*p* = 0.016). The *p*‐value of 0.016 suggests a statistically significant difference between the EBT and non‐EBT sites. For blood use feedback to clinicians or departments, 68 sites (60.7%) using EBTs reported feedback compared to 36 sites (39.3%) without EBTs, but the difference was not statistically significant (*p* = 0.723).

**TABLE 4:** Feedback and blood data availability to clinicians, traceability of blood, and number of staff in blood transfusion labs in relation to EBTs (latest year, 2023).

Question	Category	No EBTs (%)	Yes EBTs (%)	Total responses	Chi‐square (*p*‐value)	Fisher's exact test
Are data on blood use fed back to clinicians or departments?	No	15/112 (13.4%)	29/112 (25.9%)	44/112 (39.3%)	0.723	0.836
	Yes	21/112 (18.8%)	47/112 (42.0%)	68/112 (60.7%)		
Is data on blood usage available to clinicians to look up (routinely vs. not routinely)?	Not routinely	36/112 (32.1%)	65/112 (58.0%)	101/112 (90.2%)	0.016	0.016
	Routinely	0/112 (0.0%)	11/112 (9.8%)	11/112 (9.8%)		
Categories for traceability levels	≤98%	0/112 (0.0%)	5/112 (4.5%)	5/112	0.115	0.131
	98%–99%	14/112 (12.5%)	18/112 (16.1%)	32/112		
	100%	23/112 (20.5%)	52/112 (46.4%)	75/112		
Categories for number of staff	Very small (≤10)	21/113 (18.6%)	49/113 (43.4%)	70/113	0.570	0.570
	Medium (11–20)	9/113 (8.0%)	18/113 (15.9%)	27/113		
	Large (>20)	7/113 (6.2%)	9/113 (8.0%)	16/113		

Abbreviation: EBT, electronic blood transfusion.

The analysis of traceability levels showed that 100% traceability was achieved by 52 sites (46.4%) using EBTs compared to 23 sites (20.5%) without EBTs. Moderate traceability (98%–99%) was observed in 18 EBT‐using sites (16.1%) and 14 non‐EBT sites (12.5%). Lower traceability (≤98%) occurred only in five EBT‐using sites (4.5%), with no non‐EBT sites in this category. However, these differences were not statistically significant (*p* = 0.115).

The analysis of staff size in blood transfusion laboratories showed that EBT adoption was more frequently associated with services with very small staff (49 sites, 43.4%) compared to non‐EBT services in the same category (21 sites, 18.6%). For medium‐sized staff services, 18 sites (15.9%) reported the use of EBTs compared to 9 sites (8.0%) without EBTs. Among large services, nine sites (8.0%) reported using EBTs compared to seven sites (6.2%) that had not. Despite these distributional differences, no statistically significant association was found between staff size and EBT use (*p* = 0.570).

#### Intention to implement EBTs and business cases

The analysis of sites planning to implement EBTs (Table 5) showed varying levels of interest across the individual components of EBTs. For sample labelling systems, 17 sites (36.2%) indicated implementation plans, while 30 sites (63.8%) did not (*p* = 0.025). Similarly, for patient identification administration systems, 19 sites (46.3%) reported implementation plans, whereas 22 sites (53.7%) did not (*p* = 0.012).

**TABLE 5:** Intention to implement EBTs for hospitals that do not have them (latest year, 2023).

Question	No (*N*/%)	Yes (*N*/%)	Total responses^a^	Chi‐square (*p*‐value)	Fisher's exact test
Sample labelling	30 (63.83%)	17 (36.17%)	47	5.03 (0.025)	−0.03
Patient ID administration	22 (53.66%)	19 (46.34%)	41	6.29 (0.012)	−0.018
Blood fridge	6 (66.67%)	3 (33.33%)	9	8.04 (0.005)	−0.007
Remote issue	50 (73.53%)	18 (26.47%)	68	3.78 (0.052)	−0.057
Order no CDSS	64 (91.43%)	6 (8.57%)	70	10.21 (0.001)	−0.002
Order with CDSS	49 (81.67%)	11 (18.33%)	60	7.45 (0.006)	−0.008
Linkage with EHRs	37 (82.22%)	8 (17.78%)	45	9.12 (0.003)	−0.004
Linkage of records within/between hospitals	27 (61.36%)	17 (38.64%)	44	4.66 (0.031)	−0.036
Traceability	17 (58.62%)	12 (41.38%)	29	6.55 (0.010)	−0.013

Abbreviations: CDSS, clinical decision support system; EBT, electronic blood transfusion; EHR, electronic health record.

^a^ Data represent only those respondents who do not currently have the respective EBT systems implemented and have explicitly answered whether they plan to implement them or not. This is the number of hospitals that provided clear intentions regarding future implementation.

EBFs had the lowest intent, with only three sites (33.3%) planning implementation (*p* = 0.005). Similarly, remote issue systems showed limited intent, with 18 sites (26.5%) planning implementation (*p* = 0.052). Systems with CDSS showed more interest than those without, with 11 sites (18.3%) planning CDSS‐based systems and 6 sites (8.6%) planning non‐CDSS systems (*p* = 0.006 and *p* = 0.001 respectively). For traceability, 12 sites (41.4%) expressed intent, compared to 17 (58.6%) that did not (*p* = 0.010).

The analysis of business case development (Table 6) found that 58 sites (52.3%) developed business cases, while 53 (47.8%) did not. For specific EBTs, 40 sites (78.4%) included blood sample collection in their cases, with 27 (69.2%) reporting success. Blood administration was included by 44 sites (86.3%), with 30 (69.8%) achieving success.

**TABLE 6:** Business cases for EBT implementation (before or after 2021).

Question	No (*N*/%)	Yes (*N*/%)	Total (*N*)
Have you developed a business case for implementing EBTs?	53/111 (47.75%)	58/111 (52.25%)	111
Blood sample collection: Included in business case	11/51 (21.57%)	40/51 (78.43%)	51
Blood sample collection: Successful business case	12/39 (30.77%)	27/39 (69.23%)	39
Blood administration: Included in business case	7/51 (13.73%)	44/51 (86.27%)	51
Blood administration: Successful business case	13/43 (30.23%)	30/43 (69.77%)	43
Collection from blood fridges: Included in business case	3/48 (6.25%)	45/48 (93.75%)	48
Collection from blood fridges: Successful business case	9/44 (20.45%)	35/44 (79.55%)	44
Blood ordering and decision support: Included in business case	43/55 (78.18%)	12/55 (21.82%)	55
Blood ordering and decision support: Successful business case	3/10 (30.00%)	7/10 (70.00%)	10

Abbreviation: EBT, electronic blood transfusion.

Collection from blood fridges showed the highest business case development rate at 45 sites (93.8%), with 35 (79.6%) achieving success. Blood ordering and decision support had lower rates, with 12 sites (21.8%) including it in the business case and 7 (70.0%) reporting success. These findings highlight higher engagement in business case development for simpler systems compared to more complex components like CDSS.

## DISCUSSION

This study provides an updated overview of EBT system use across NHS hospitals in England, highlighting both progress and persistent challenges. While foundational EBT components like blood fridges are used by 57.5% of surveyed sites (65 sites), advanced technologies such as electronic blood ordering systems incorporating CDSS (14.2%, 16 sites) and EBFs with remote blood issue (8.0%, 9 sites) remain less widely used. This disparity underscores significant gaps in the adoption of electronic transfusion systems and emphasises the need for targeted efforts to promote them, as already recommended in the report of the infected blood inquiry.^15^

The prevalent use of electronic blood fridges without remote issue capabilities reflects an initial emphasis on enhancing blood stock management and improving blood traceability and is the simplest EBT for hospitals to implement. Yet, the slower uptake of more complex systems, such as electronic PID and remote ordering with CDSS, suggests challenges in obtaining the support and resources. These EBT systems are crucial for avoiding errors resulting in wrong blood transfusions and reducing inappropriate blood use, but require more extensive changes to hospital procedures and training.^1^, ^6^

Financial constraints were identified as the most significant barrier to EBT implementation, with 70.7% of respondents citing insufficient funding from senior management as a very high or high obstacle (*p* < 0.001). This aligns with the recommendation from the infected blood inquiry,^15^ which emphasised prioritising funding for electronic transfusion systems. Additionally, a lack of robust health economic evidence was highlighted as a key factor hindering adoption. Without this robust cost‐effectiveness evidence, hospitals face challenges in securing the necessary resources and leadership support. As highlighted in the latest NICE guideline on blood transfusion,^26^ stronger health economic evidence is needed to demonstrate that EBT interventions not only enhance patient safety and improve resource efficiency^1^, ^3^ but also represent a cost‐effective investment.

Traceability is a critical component of transfusion safety, with 46.4% of EBT‐using sites reporting 100% traceability compared to 20.5% of non‐EBT sites, although this difference was not statistically significant. These findings underscore the need for continued efforts to enhance traceability through effective system implementation and operational consistency.^5^, ^6^

Staffing levels appeared less critical to EBT adoption, as similar implementation rates were observed across very small, medium and large blood transfusion labs. While good inventory performance is often attributed to advanced models and algorithms, evidence suggests that skilled, regularly trained and experienced transfusion laboratory staff often play a more critical role.^10^

Intentions to implement EBTs varied in our survey. Foundational systems like sample labelling (36.2%) and patient identification (46.3%) were prioritised, while advanced systems like CDSS‐based ordering (18.3%) had limited interest. This trend may reflect concerns about cost, complexity and a lack of economic evidence for advanced systems. Demonstrating the cost‐effectiveness of EBT technologies is crucial for securing funding and leadership support.

Business case development emerged as a critical enabler of EBT implementation. Over half of the sites developed business cases for EBTs, with simpler components like EBFs achieving higher success rates (79.6%) compared to advanced systems like CDSS (70.0%). These findings highlight the importance of clearly demonstrating the clinical and economic benefits of EBTs to justify investments. The infected blood inquiry emphasised this need, recommending dedicated funding, robust health economic evaluations and a framework to track outcomes for blood recipients.^15^

Technical challenges related to system integration highlight the critical need for robust IT support and infrastructure upgrades. Seamless integration of EBT systems into existing hospital IT environments is essential to maximise their potential and ensure reliable performance.^27^, ^28^ This integration is not merely a technical necessity but also a strategic investment with the potential to yield long‐term benefits in operational efficiency and patient safety.^18^, ^20^, ^29^

In conclusion, while EBT systems offer significant potential to enhance transfusion practices, their varied adoption and the barriers to adoption identified in this study underscore the necessity for targeted strategies to promote their implementation. Generating robust health economic evidence and providing funding support for hospitals are pivotal steps towards achieving widespread adoption and realising the full benefits of EBT systems in the NHS.

### Limitations

This study has several limitations. First, it relies on self‐reported data, which may introduce bias and limit the accuracy of reported EBT adoption and effectiveness. The response rate of 55.6% raises the possibility of non‐response bias; however, it exceeds typical survey response rates. Additional analyses using publicly available data on organisational and geographical characteristics found no significant differences between responding and non‐responding sites, supporting the sample's representativeness.

Second, respondents selected barriers and priorities from a predefined list, which may have constrained their ability to report context‐specific issues. Third, the survey did not capture hospital‐specific factors that could influence EBT adoption and outcomes. Finally, as the study was limited to hospitals in England, the findings may not be directly applicable to other parts of the UK with different regulatory or funding structures.

## CONCLUSION

This study shows that, while some EBT systems, like electronic blood fridges, are widely used, advanced systems like CDSS are less common. Financial barriers and a lack of robust health economic evidence hinder broader adoption. Targeted investments and stronger evidence on cost‐effectiveness are needed to overcome these challenges and improve transfusion safety and efficiency in hospitals across England.

## AUTHOR CONTRIBUTIONS

F. Tomini, S. Staples, H. Evans, M. F. Murphy and S. J. Stanworth contributed to the design of the data collection tool. All authors contributed to further developing and piloting the data collection tool. F. Tomini wrote the first draft of the manuscript. All authors contributed to the final version.

## FUNDING INFORMATION

This publication is supported by the National Institute for Health and Care Research (NIHR) Blood and Transplant Research Unit in Data Driven Transfusion Practice (NIHR203334). The views expressed are those of the author(s) and not necessarily those of the NIHR or the Department of Health and Social Care.

## CONFLICT OF INTEREST STATEMENT

The authors declare no conflicts of interest.

## ETHICS APPROVAL STATEMENT

Not applicable.

## PERMISSION TO REPRODUCE MATERIAL FROM OTHER SOURCES

Not applicable. This study does not include any material reproduced from other sources.

## Supporting information


Table S1.

Table S2.

Table S3.

Table S4.

Table S5.

Table S6.

Table S7.

## Data Availability

The data supporting the findings of this study are available from the corresponding author upon reasonable request.

## References

[1] Swart N , Morris S , Murphy MF . Economic value of clinical decision support allied to direct data feedback to clinicians: blood usage in haematology. Vox Sang. 2020;115(4):293–302. 32034773 10.1111/vox.12880

[2] Verlicchi F , Pacilli P , Bragliani A , Rapuano S , Dini D , Vincenzi D . Electronic remote blood issue combined with a computer‐controlled, automated refrigerator for major surgery in operating theatres at a distance from the transfusion service. Transfusion. 2018;58(2):372–378. 29193169 10.1111/trf.14418

[3] Staples S , Staves J , Davies J , Polley N , Boyd JS , Lukas M , et al. Electronic remote blood issue supports efficient and timely supply of blood and cost reduction: evidence from five hospitals at different stages of implementation. Transfusion. 2019;59(5):1683–1691. 30860601 10.1111/trf.15231

[4] Murphy MF , Jayne Addison J , Poles D , Dhiman P , Bolton‐Maggs P . Electronic identification systems reduce the number of wrong components transfused. Transfusion. 2019;59(12):3601–3607. 31584694 10.1111/trf.15537

[5] Kaufman RM , Dinh A , Cohn CS , Fung MK , Gorlin J , Melanson S , et al. Electronic patient identification for sample labeling reduces wrong blood in tube errors. Transfusion. 2019;59(3):972–980. 30549289 10.1111/trf.15102

[6] Hibbs SP , Nielsen ND , Brunskill S , Doree C , Yazer MH , Kaufman RM , et al. The impact of electronic decision support on transfusion practice: a systematic review. Transfus Med Rev. 2015;29(1):14–23. 25535095 10.1016/j.tmrv.2014.10.002

[7] Hibbs SP , Noel S , Miles D , Staves J , Murphy MF . The impact of electronic decision support and electronic remote blood issue on transfusion practice. Transfus Med. 2014;24(5):274–279. 25186089 10.1111/tme.12149

[8] Sandler SG , Langeberg A , Dohnalek L . Bar code technology improves positive patient identification and transfusion safety. Dev Biol (Basel). 2005;120:19–24. 16050151

[9] Forest SK , Shirazi M , Wu‐Gall C , Stotler BA . The impact of an electronic ordering system on blood bank specimen rejection rates. Am J Clin Pathol. 2017;147(1):105–109. 28158445 10.1093/ajcp/aqw204

[10] Stanger SH , Yates N , Wilding R , Cotton S . Blood inventory management: hospital best practice. Transfus Med Rev. 2012;26(2):153–163. 22018647 10.1016/j.tmrv.2011.09.001

[11] Frank SM , Oleyar MJ , Ness PM , Tobian AAR . Reducing unnecessary preoperative blood orders and costs by implementing an updated institution‐specific maximum surgical blood order schedule and a remote electronic blood release system. Anesthesiology. 2014;121(3):501–509. 24932853 10.1097/ALN.0000000000000338 PMC4165815

[12] Horck S , Fahy N , Greenhalgh T . Implementation challenges of electronic blood transfusion safety systems: lessons from an international, multi‐site comparative case study. Transfus Med. 2025;35(1):48–59. 39252454 10.1111/tme.13095 PMC11833216

[13] Ruutiainen H , Holmström AR , Kunnola E , Kuitunen S . Use of computerized physician order entry with clinical decision support to prevent dose errors in pediatric medication orders: a systematic review. Paediatr Drugs. 2024;26(2):127–143. 38243105 10.1007/s40272-023-00614-6 PMC10891203

[14] Morrison AP , Tanasijevic MJ , Goonan EM , Lobo MM , Bates MM , Lipsitz SR , et al. Reduction in specimen labeling errors after implementation of a positive patient identification system in phlebotomy. Am J Clin Pathol. 2010;133(6):870–877. 20472844 10.1309/AJCPC95YYMSLLRCX

[15] Infected Blood Inquiry: The Report. Presented to Parliament pursuant to section 26 of the Inquiries Act 2005. HC 569‐I. 2024.

[16] NHS Blood and Transplant . Blood stocks management scheme inventory practice survey. Bristol, UK: NHS Blood and Transplant; 2021.

[17] NHS Digital . NHS in Numbers Today [Internet]. 2025. Available from: https://www.england.nhs.uk/statistics/

[18] Evans HG , Murphy MF , Foy R , Dhiman P , Green L , Kotze A , et al. Harnessing the potential of data‐driven strategies to optimise transfusion practice. Br J Haematol. 2024;204(1):74–85. 37964471 10.1111/bjh.19158

[19] Sellen KM , Jovanovic A , Perrier L , Chignell M . Systematic review of electronic remote blood issue. Vox Sang. 2015;109(1):35–43. 25827223 10.1111/vox.12240

[20] Staples S , Salisbury RA , King AJ , Polzella P , Bakhishli G , Staves J , et al. How do we use electronic clinical decision support and feedback to promote good transfusion practice. Transfusion. 2020;60(8):1658–1665. 32643142 10.1111/trf.15864

[21] Yazer MH , Deandrade DS , Triulzi DJ , Wisniewski MK , Waters JH . Electronic enhancements to blood ordering reduce component waste. Transfusion. 2016;56(3):564–570. 26559520 10.1111/trf.13399

[22] Kelly SL , Reed MJ , Innes CJ , Manson L . A review of blood component usage in a large UK emergency department after implementation of simple measures. Emerg Med J. 2013;30(10):842–845. 23144079 10.1136/emermed-2012-201747

[23] Fisher RA . Statistical methods for research workers. In: Kotz S , Johnson NL , editors. Breakthroughs in statistics: methodology and distribution. New York: Springer New York; 1992. p. 66–70.

[24] Campbell MJ . Statistics at square one ‐ 12th edition. New York: John Wiley & Sons; 2021.

[25] NHS England . Bed Availability and Occupancy Data – Overnight [Internet]. 2024 [cited 2024 Nov 3]. Available from: https://www.england.nhs.uk/statistics/statistical‐work‐areas/bed‐availability‐and‐occupancy/bed‐data‐overnight/

[26] NICE . Guideline on blood transfusion NG24 (updated 2023). England: National Institute of Health and Care Excellence; 2015. 32813480

[27] Stoffel M , Leu MG , Barry D , Hrachovec J , Saifee NH , Migita DS , et al. Optimizing electronic blood ordering and supporting administration workflows to improve blood utilization in the pediatric hospital setting. Transfusion. 2023;63(12):2328–2340. 37942518 10.1111/trf.17587

[28] Reed MJ , Kelly SL , Beckwith H , Innes CJ , Manson L . Successful implementation of strategies to transform emergency department transfusion practice. BMJ Qual Improv Rep. 2013;2(1):u201055.w690. 10.1136/bmjquality.u201055.w690 PMC4652721 26734190

[29] Li N , Arnold DM , Down DG , Barty R , Blake J , Chiang F , et al. From demand forecasting to inventory ordering decisions for red blood cells through integrating machine learning, statistical modeling, and inventory optimization. Transfusion. 2022;62(1):87–99. 34784053 10.1111/trf.16739

