# Supplementary material for: Unlocking the potential of electronic blood transfusion systems: Implementation insights from NHS hospitals in England
Source: Br J Haematol. 2025 Jun 10;207(1):235–43. doi: 10.1111/bjh.20198 (PMC12234281; doi:10.1111/bjh.20198)
Supplement: Supplementary file 1 — Table S1. [file BJH-207-235-s002.docx]

Table S1. Respondents vs. Non-Respondents by Hospital Service Status

|  | **Non-Respondents(N, %)** | **Respondents(N, %)** | **Statistical Tests** |
| --- | --- | --- | --- |
| No: MTC | 83 (46.37%) | 96 (53.63%) | Pearson χ² (1) = 1.613, p = 0.204  Fisher’s Exact (2-sided) = 0.221 |
| Yes: MTC | 9 (33.33%) | 18 (66.67%) |  |
|  |  |  |  |
| No: Cardiac Centre | 82 (45.81%) | 97 (54.19%) | Pearson χ² (1) = 0.731, p = 0.393  Fisher’s Exact (2-sided) = 0.415 |
| Yes: Cardiac Centre | 10 (37.04%) | 17 (62.96%) |  |
|  |  |  |  |
| No: ECMO Centre | 91 (45.50%) | 109 (54.50%) | Pearson χ² (1) = 1.960, p = 0.162  Fisher’s Exact (2-sided) = 0.228 |
| Yes: ECMO Centre | 1 (16.67%) | 5 (83.33%) |  |
|  |  |  |  |
| No: HEMS Status | 87 (46.28%) | 101 (53.72%) | Pearson χ² (1) = 2.275, p = 0.132  Fisher’s Exact (2-sided) = 0.146 |
| Yes: HEMS Status | 5 (27.78%) | 13 (72.22%) |  |
|  |  |  |  |
| No: BMT Centre | 83 (45.11%) | 101 (54.89%) | Pearson χ²(1) = 0.1402, p=0.708  Fisher’s Exact (2-sided) = 0.822 |
| Yes: BMT Centre | 9 (40.91%) | 13 (59.09%) |  |
|  |  |  |  |
| Total | 92 (44.66%) | 114 (55.34%) |  |

Note: The sites may or may not have one of the following services: Major Trauma Centre (MTC), Cardiac Centre, Extracorporeal Membrane Oxygenation (ECMO), Helicopter Emergency Medical Service (HEMS), and Bone Marrow Transplant (BMT).
