# Supplementary material for: Unlocking the potential of electronic blood transfusion systems: Implementation insights from NHS hospitals in England
Source: Br J Haematol. 2025 Jun 10;207(1):235–43. doi: 10.1111/bjh.20198 (PMC12234281; doi:10.1111/bjh.20198)
Supplement: Supplementary file 2 — Table S2. [file BJH-207-235-s006.docx]

Table S2: Respondents vs. Non-Respondents by Trust Size (Bed Capacity)

| **Trust Size (Bed Capacity)** | **Non-Respondents(N, %)** | **Respondents(N, %)** | **Statistical Tests** |
| --- | --- | --- | --- |
| Small Capacity (<300) | 6 (35.29%) | 11 (64.71%) | Pearson χ² (1) = 1.960, p = 0.162  Fisher’s Exact (2-sided) = 0.228  Fisher’s Exact (1-sided) = 0.164 |
| Medium Capacity (300–999) | 41 (42.71%) | 55 (57.29%) |  |
| Large Capacity (1,000–2,000) | 38 (46.34%) | 44 (53.66%) |  |
| Very Large Capacity (>2,000) | 7 (63.64%) | 4 (36.36%) |  |
| Total | 92 (44.66%) | 114 (55.34%) |  |

Note: The number of beds is reported per corresponding NHS trust (one NHS Trust may have one or multiple sites reporting). Capacity categories are defined as Small (<300), Medium (300–999), Large (1,000–2,000), and Very Large (>2,000).
