# Supplementary material for: Unlocking the potential of electronic blood transfusion systems: Implementation insights from NHS hospitals in England
Source: Br J Haematol. 2025 Jun 10;207(1):235–43. doi: 10.1111/bjh.20198 (PMC12234281; doi:10.1111/bjh.20198)
Supplement: Supplementary file 3 — Table S3. [file BJH-207-235-s004.docx]

Table S3: Respondents vs. Non-Respondents by NHSBT SHU (Supplying Centre)

| **NHSBT SHU** | **Non-Respondents(N, %)** | **Respondents(N, %)** | **Statistical Tests** |
| --- | --- | --- | --- |
| Barnsley | 15 (46.88%) | 17 (53.12%) | Pearson χ² (13)=18.1720; p=0.151  Fisher's exact=0.148 |
| Basildon | 8 (72.73%) | 3 (27.27%) |  |
| Birmingham | 13 (56.52%) | 10 (43.48%) |  |
| Cambridge | 1 (11.11%) | 8 (88.89%) |  |
| Colindale | 11 (52.38%) | 10 (47.62%) |  |
| Filton | 4 (50.00%) | 4 (50.00%) |  |
| Lancaster | 4 (57.14%) | 3 (42.86%) |  |
| Liverpool | 2 (18.18%) | 9 (81.82%) |  |
| Manchester | 5 (35.71%) | 9 (64.29%) |  |
| Newcastle | 5 (41.67%) | 7 (58.33%) |  |
| Oxford | 4 (36.36%) | 7 (63.64%) |  |
| Plymouth | 0 (0.00%) | 5 (100.00%) |  |
| Southampton | 4 (44.44%) | 5 (55.56%) |  |
| Tooting | 16 (48.48%) | 17 (51.52%) |  |
| Total | 92 (44.66%) | 114 (55.34%) |  |

Note: NHSBT SHU refers to the NHS Blood and Transplant’s Stock Holding Unit (SHU), which manages local storage and distribution of blood components to the closest hospitals.
