# Supplementary material for: Unlocking the potential of electronic blood transfusion systems: Implementation insights from NHS hospitals in England
Source: Br J Haematol. 2025 Jun 10;207(1):235–43. doi: 10.1111/bjh.20198 (PMC12234281; doi:10.1111/bjh.20198)
Supplement: Supplementary file 4 — Table S4. [file BJH-207-235-s005.docx]

Table S4: Respondents vs. Non-Respondents by SHU Distance (Miles)

| **SHU Distance Category** | **Non-Respondents(N, %)** | **Respondents(N, %)** | **Statistical Tests** |
| --- | --- | --- | --- |
| ≤10 miles | 24 (40.00%) | 36 (60.00%) | Pearson χ² (3) = 1.377, p = 0.711  Fisher’s Exact (2-sided) = 0.722 |
| 11–25 miles | 25 (47.17%) | 28 (52.83%) |  |
| 26–50 miles | 30 (49.18%) | 31 (50.82%) |  |
| >50 miles | 13 (40.62%) | 19 (59.38%) |  |
| Total | 92 (44.66%) | 114 (55.34%) |  |

Note: Distance groups are determined by the distance to the nearest NHS Blood and Transplant’s Stock Holding Unit (SHU): ≤10 miles, 11–25 miles, 26–50 miles, >50 miles.
