# Supplementary material for: Unlocking the potential of electronic blood transfusion systems: Implementation insights from NHS hospitals in England
Source: Br J Haematol. 2025 Jun 10;207(1):235–43. doi: 10.1111/bjh.20198 (PMC12234281; doi:10.1111/bjh.20198)
Supplement: Supplementary file 5 — Table S5. [file BJH-207-235-s007.docx]

Table S5. Number of EBTs components implemented for each of the implementation patterns (latest year =2023)

| Variable | **Implementation number in each pathway** | | **Sample taking and labelling** | | | **Patient identification and administration of blood** | | | **Blood fridges** | | | **Remote issue** | | | **Electronic blood ordering without clinical decision support** | | | **Electronic blood ordering without clinical decision support** | | | **Linkage with EHRs** | | | **Linkage of records within/between hospitals** | | | **Traceability Procedures** | | |
| --- | --- | --- | --- | --- | --- | --- | --- | --- | --- | --- | --- | --- | --- | --- | --- | --- | --- | --- | --- | --- | --- | --- | --- | --- | --- | --- | --- | --- | --- |
|  | N | % | % | % | N | | % | N | | % | N | | % | N | | % | N | | % | N | | % | N | | % | N | | % |  |
| Hospitals with: | | | | | | | | | | | | | | | | | | | | | | | | | | | | | |
| One EBT | 17 | 15.0% | 0 | 0.0% | 0 | | 0.0% | 16 | | 14.2% | 0 | | 0.0% | 0 | | 0.0% | 0 | | 0.0% | 0 | | 0.0% | 1 | | 0.9% | 1 | | 0.9% |  |
| Two EBTs | 9 | 8.0% | 2 | 1.8% | 0 | | 0.0% | 8 | | 7.1% | 0 | | 0.0% | 0 | | 0.0% | 0 | | 0.0% | 1 | | 0.9% | 5 | | 4.4% | 2 | | 1.8% |  |
| Three EBTs | 15 | 13.3% | 0 | 0.0% | 2 | | 1.8% | 13 | | 11.5% | 1 | | 0.9% | 0 | | 0.0% | 1 | | 0.9% | 7 | | 6.2% | 6 | | 5.3% | 8 | | 7.1% |  |
| Four EBTs | 17 | 15.0% | 9 | 8.0% | 11 | | 9.7% | 15 | | 13.3% | 1 | | 0.9% | 0 | | 0.0% | 3 | | 2.7% | 6 | | 5.3% | 8 | | 7.1% | 15 | | 13.3% |  |
| Five EBTs | 16 | 14.2% | 10 | 8.8% | 15 | | 13.3% | 14 | | 12.4% | 3 | | 2.7% | 2 | | 1.8% | 2 | | 1.8% | 10 | | 8.8% | 8 | | 7.1% | 15 | | 13.3% |  |
| Six EBTs | 7 | 6.2% | 5 | 4.4% | 6 | | 5.3% | 6 | | 5.3% | 1 | | 0.9% | 1 | | 0.9% | 5 | | 4.4% | 6 | | 5.3% | 6 | | 5.3% | 7 | | 6.2% |  |
| Seven EBTs | 6 | 5.3% | 5 | 4.4% | 5 | | 4.4% | 5 | | 4.4% | 2 | | 1.8% | 0 | | 0.0% | 5 | | 4.4% | 5 | | 4.4% | 3 | | 2.7% | 5 | | 4.4% |  |
| Eight EBTs | 1 | 0.9% | 1 | 0.9% | 1 | | 0.9% | 1 | | 0.9% | 1 | | 0.9% | 0 | | 0.0% | 1 | | 0.9% | 1 | | 0.9% | 1 | | 0.9% | 1 | | 0.9% |  |
| Nine EBTs | 1 | 0.9% | 1 | 0.9% | 1 | | 0.9% | 1 | | 0.9% | 1 | | 0.9% | 1 | | 0.9% | 1 | | 0.9% | 1 | | 0.9% | 1 | | 0.9% | 1 | | 0.9% |  |
| Missing | 2 | 1.8% | 0 | 0.0% | 0 | | 0.0% | 0 | | 0.0% | 0 | | 0.0% | 0 | | 0.0% | 0 | | 0.0% | 0 | | 0.0% | 0 | | 0.0% | 0 | | 0.0% |  |
