# Supplementary material for: Unlocking the potential of electronic blood transfusion systems: Implementation insights from NHS hospitals in England
Source: Br J Haematol. 2025 Jun 10;207(1):235–43. doi: 10.1111/bjh.20198 (PMC12234281; doi:10.1111/bjh.20198)
Supplement: Supplementary file 6 — Table S6. [file BJH-207-235-s001.docx]

Table S6. Year of implementation of different EBTs components and frequency

| **Implementation**  (Year/ N) | **Sample taking and labelling** | **Patient identification and administration of blood** | **Blood fridges** | **Remote issue** | **Electronic blood ordering without clinical decision support** | **Electronic blood ordering without clinical decision support** | **Linkage with EHRs** | **Linkage of records within/between hospitals** | **Traceability Procedures** |
| --- | --- | --- | --- | --- | --- | --- | --- | --- | --- |
| First year of implementation | 2001 | 1998 | 1998 | 2004 | 2019 | 2008 | 2005 | 2003 | 1998 |
| Q1 (25%) | 2015 | 2012 | 2009 | 2014 | 2019 | 2015 | 2015 | 2014 | 2009 |
| Q3 (75%) | 2022 | 2021 | 2019 | 2020 | 2022 | 2023 | 2022 | 2022 | 2019 |
| Median (50%) | 2019 | 2019 | 2014 | 2018 | 2022 | 2020 | 2020 | 2019 | 2015 |
| Mode (year) | 2022 | 2019 | 2019 | 2021 | 2022 | 2023 | 2023 | 2023 | 2019 |
