# Supplementary material for: Unlocking the potential of electronic blood transfusion systems: Implementation insights from NHS hospitals in England
Source: Br J Haematol. 2025 Jun 10;207(1):235–43. doi: 10.1111/bjh.20198 (PMC12234281; doi:10.1111/bjh.20198)
Supplement: Supplementary file 7 — Table S7. [file BJH-207-235-s003.docx]

Table S7. The estimated proportion of all transfusions throughout the hospital that use the particular EBTs (latest year = 2023)

|  |  | **Implemented for the % of total transfusions in the hospital:** | | | |
| --- | --- | --- | --- | --- | --- |
| **Variable** | **Not Implemented** | **0 - 20%*** | **20 - 50%** | **>50%** | **100%** |
| **Sample Taking and Labelling** | 84/113 (74.3%) | 0/113 (0%) | 6/113 (5.3%) | 10/113 (8.8%) | 13/113 (11.5%) |
| **Patient Identification and Administration** | 76/113 (67.3%) | 2/113 (1.8%) | 5/113 (4.4%) | 9/113 (8.0%) | 21/113 (18.6%) |
| **Blood Fridges** | 48/113 (42.5%) | 0/113 (0%) | 0/113 (0%) | 10/113 (8.8%) | 55/113 (48.7%) |
| **Remote Issue** | 104/113 (92.0%) | 0/113 (0%) | 2/113 (1.8%) | 4/113 (3.5%) | 3/113 (2.7%) |
| **Electronic Blood Ordering without CDSS** | 110/113 (97.3%) | 1/113 (0.9%) | 0/113 (0%) | 0/113 (0%) | 2/113 (1.8%) |
| **Electronic Blood Ordering with CDSS** | 97/113 (85.8%) | 0/113 (0%) | 1/113 (0.9%) | 2/113 (1.8%) | 13/113 (11.5%) |
| **Linkage with EHRs** | 81/113 (71.7%) | 0/113 (0%) | 0/113 (0%) | 3/113 (2.7%) | 29/113 (25.7%) |
| **Linkage of Records Within/Between Hospitals** | 80/113 (70.8%) | 0/113 (0%) | 0/113 (0%) | 1/113 (0.9%) | 32/113 (28.3%) |
| **Traceability Procedures** | 66/113 (58.4%) | 0/113 (0%) | 2/113 (1.8%) | 7/113 (6.2%) | 38/113 (33.6%) |

Note: * - This indicates the number of sites that reported specific percentages for transfusions across the hospital that utilise the particular EBTs.
